# Identification of new major histocompatibility complex-B Haplotypes in Bangladesh native chickens

**DOI:** 10.5713/ab.23.0295

**Published:** 2024-02-23

**Authors:** Thisarani Kalhari Ediriweera, Prabuddha Manjula, Jaewon Kim, Jin Hyung Kim, Seonju Nam, Minjun Kim, Eunjin Cho, Mohammad Shamsul Alam Bhuiyan, Md. Abdur Rashid, Jun Heon Lee

**Affiliations:** 1 Department of Bio-AI Convergence, Chungnam National University, Daejeon 34134, Korea; 2 Department of Animal Science, Uva Wellassa University, Badulla 90000, Sri Lanka; 3 Division of Animal and Dairy Science, Chungnam National University, Daejeon 34134, Korea; 4 Department of Animal Breeding and Genetics, Bangladesh Agricultural University, Mymensingh-2202, Bangladesh; 5 Poultry Production Research Division, Bangladesh Livestock Research Institute, Dhaka-1341, Bangladesh

**Keywords:** Bangladesh Native Chicken, Haplotypes, Phylogeny, Kompetitive Allele Specific Polymerase Chain Reaction (PCR), Major Histocompatibility Complex-B

## Abstract

**Objective:**

The major histocompatibility complex in chicken demonstrates a great range of variations within varities, breeds, populations and that can eventually influence their immuneresponses. The preset study was conducted to understand the major histocompatibility complex-B (MHC-B) variability in five major populations of Bangladesh native chicken: Aseel, Hilly, Junglefowl, Non-descript Deshi, and Naked Neck.

**Methods:**

These five major populations of Bangladesh native chicken were analyzed with a subset of 89 single nucleotide polymorphisms (SNPs) in the high-density MHC-B SNP panel and Kompetitive Allele-Specific polymerase chain reaction genotyping was applied. To explore haplotype diversity within these populations, the results were analyzed both manually and computationally using PHASE 2.1 program. The phylogenetic investigations were also performed using MrBayes program.

**Results:**

A total of 136 unique haplotypes were identified within these five Bangladesh chicken populations, and only one was shared (between Hilly and Naked Neck). Phylogenetic analysis showed no distinct haplotype clustering among the five populations, although they were shared in distinct clades; notably, the first clade lacked Naked Neck haplotypes.

**Conclusion:**

The present study discovered a set of unique MHC-B haplotypes in Bangladesh chickens that could possibly cause varied immune reponses. However, further investigations are required to evaluate their relationships with global chicken populations.

## INTRODUCTION

Globally, native chickens help to enhance genetic diversity among chicken breeds, but they are at risk of losing their unique valuable genotypes because of intensive selection and breeding [1]. Although native chickens are not likely to be viable on an industrial scale, their desirable characteristics include unique adaptations for local conditions, high meat quality, and disease resistance, encouraging contemporary geneticists to investigate their genetic makeup and diversity [2,3].

Indigenous chickens in Bangladesh, often referred to as “Deshi chickens,” constitute a cluster of native chickens: Aseel (AS), Autosomal Dwarf, Full-Feathered, Hilly (HI), Junglefowl (JF), Naked Neck (NN), Non-Descript Deshi (ND), and Tiger [4–6]. These chickens are known for their superior geographical adaptability and high disease resistance [4].

Since the AS, HI, JF, ND, and NN breeds are important chicken genetic resources [5], we chose them as the study populations. AS is a game bird found mostly in Brahmanbaria [7], whereas HI is mostly reared for food (both meat and eggs) in hilly regions of Bangladesh [8]. JF mostly inhabits high-elevation forests and the jungles of Madhupur and Rajendrapur [9]. NN, the best egg producer [10], is distributed throughout the country [11]. ND, a native breed selected for systematic breeding programs, is available in most non-hilly areas of Bangladesh [9,12].

Considering this high disease resistance, evaluations of their immunity-related genes at the molecular level can help to understand their genetic diversity and specificity compared with known counterparts. From this perspective, studies are currently investigating the major histocompatibility complex (MHC) or MHC region, which is responsible for immunogenesis in the chicken [13,14]. The MHC region contains the MHC-Y and MHC-B sub-regions, which are separated by a GC-rich region [15]. The MHC-B region was identified first [16]; it is the most studied [17] and most comprehensively mapped [18] sub-region. Fulton et al [19] developed a single nucleotide polymorphism (SNP) panel representing the MHC-B region to time- and cost-effectively identify the genetic diversity. This SNP panel is used to evaluate MHC-B diversity in various chicken breeds, along with the LEI0258 microsatellite marker [19–25].

In this study, we genotyped the MHC-B regions of the AS, HI, JF, ND, and NN populations of Bangladesh native chickens [5] using a subset of 89 SNPs from the MHC-B SNP panel [19]. We then compared the results with known MHC-B haplotypes of global chicken breeds.

## MATERIALS AND METHODS

### Study populations and genomic DNA extraction

Institutional Animal Care and Use Committee (IACUC) reviewed the total experimental protocol and approved for this experiment (Approval number: BLRI-PCUC-003). Five Bangladesh chicken populations were chosen as study populations: AS, HI, JF, ND, and NN; 15, 20, 17, 20, and 19 birds from the respective populations were included in the experiment. Blood samples were collected from the wing vein of each individual and stored in FTA cards as dried blood spots. Then, genomic DNA was extracted from the FTA card blood spots using the PrimePrep genomic DNA extraction kit (Genetbio, Daejeon, Korea), following the manufacturer’s instructions. All sample were included in the sample used in Rashid et al [12].

### MHC-B SNP genotyping

All sampled individuals were genotyped using a subset of 89 SNPs in the high-density MHC-B SNP panel developed by Fulton et al [19]. For genotyping, the fluorescence-based (FAM and HEX) Single-Plex Kompetitive Allele-Specific polymerase chain reaction method was used.

### Haplotype identification, nomenclature, and phylogeny

Haplotypes were identified using both manual and computational approaches. The manual method followed published procedures [19,20]. For the computational approach, PHASE 2.1 software was utilized; its MS model was used for the analysis with 1,000 iterations and 1,000 burn-ins [26]. The results from both methods were compared to ensure the absence of discrepancies.

The haplotypes published in similar studies [20–25] and in the current study share 89 SNPs (from MHC0J6 to MHC178), excluding SNP MHC065, which was not identified in the present study. Because the MHC065 was not genotyped in the Bangladesh chicken population. The current haplotypes could not be directly compared with the published haplotypes for nomenclature purposes. Accordingly, the haplotypes obtained here were considered novel haplotypes and named based on the method described by Manjula et al [24]. Furthermore, the novel haplotypes were prefixed with “BAN” to indicate that they were identified in Bangladesh native chickens.

Phylogenetic analysis was conducted using the obtained BAN haplotypes using 89 SNPs only. The haplotypes were aligned using Clustal Omega, and a phylogenetic tree was generated using Mr. Bayes in Geneious Prime software (Geneious Prime 2023.0.4); BSNP-Kr01 was regarded as the outgroup, which was the novel haplotype identified in Korean native chicken in the previous research [23].

### Comparison of Bangladesh chicken MHC-B haplotypes with global BSNP haplotypes

Since SNP MHC065 was not present in Bangladesh chickens, all haplotypes differed from global BSNP haplotypes (obtained using 90 SNPs). These included the Finnish Landrace [20], USA Commercial Silkie [25], Argentinian ‘Campero-Inta’ [21], Korean native chicken [23], and Sri Lankan indigenous chicken [24]. Because of differences in SNP numbers, direct comparisons of Bangladesh haplotypes with global haplotypes would have been difficult unless all global haplotypes were reconstructed using 89 SNPs. However, we suspected that all global haplotypes were constructed from birds homozygous for both haplotypes; accordingly, the MHC065 alleles of the global haplotypes were excluded, and we created a new set of global haplotypes with 89 alleles to use in comparisons with Bangladesh chickens.

## RESULTS AND DISCUSSION

### Bangladesh MHC-B SNP haplotypes

**Figure 1:**
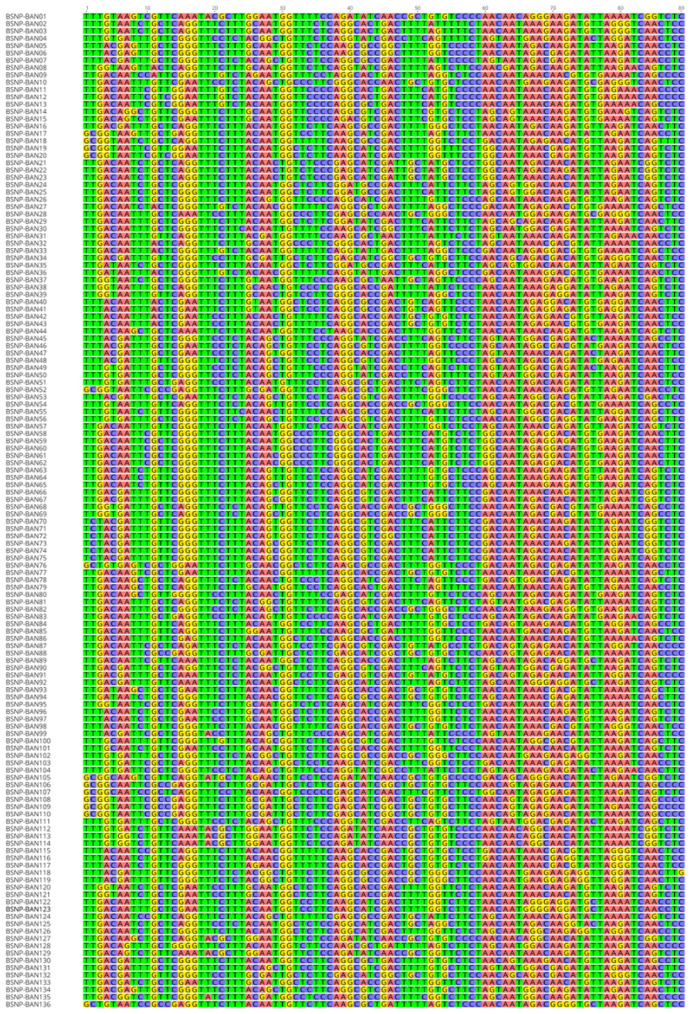
BSNP haplotypes of Bangladesh chickens obtained using an 89 SNP subset of the MHC-B SNP panel. The numbers at the top are SNP numbers.

Analysis of all five Bangladesh chicken populations together revealed 137 haplotypes: 20, 32, 24, 34, and 27 from the AS, HI, JF, ND, and NN populations, respectively. Only one haplotype was shared: BSNP-BAN49, shared by the HI and NN populations. Accordingly, all haplotypes obtained for the AS, JF, and ND populations were unique. Table 1 summarizes the obtained haplotypes; full-length haplotypes with specific haplotype names are shown in Figure 1.

### Phylogenetic analysis

**Figure 2:**
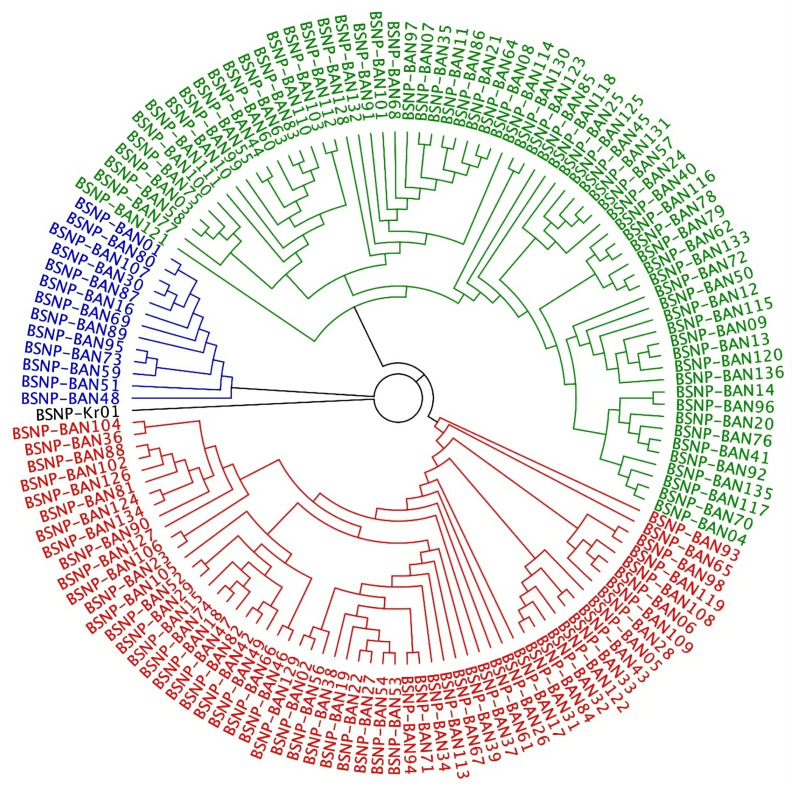
Bayesian approach-based circular cladogram for BSNP haplotypes of Bangladesh chickens.

Figure 2 shows the cladogram of the obtained haplotypes of the five populations; they formed three major clades (highlighted in blue, green, and red), with BSNP-Kr01 as the outgroup. Because the BAN haplotypes contained only 89 SNPs, they could not be directly compared with the BSNP-Kr01 standard haplotype, which was originally identified by Manjula et al [23]. Therefore, the BSNP-Kr01 haplotype was redeveloped by removing SNP MHC065 from its raw dataset. The redeveloped version of BSNP-Kr01 (with 89 SNPs) was then used as the outgroup (in black) to avoid possible bias.

Analysis of the results did not reveal a distinct pattern. Instead, each clade (except the blue clade) contained haplotypes from each of the five populations. The blue clade contained haplotypes from the AS, HI, ND, JF, but it lacks the haplotypes from NN populations. Although many highly specific haplotypes were included in the analysis, they did not phylogenetically cluster according to populations.

We speculated that it would be better to compare the BAN haplotypes with the published standard MHC haplotypes of various global chicken breeds/populations [20–25]. The comparison of the Bangladesh BSNP haplotypes with the aforementioned populations revealed that no Bangladesh chicken haplotypes matched the reported haplotypes, suggesting that all haplotypes were unique and population-specific. However, upon removal of the MHC065 allele from the reported haplotypes, some standard haplotypes that only differed at this SNP became identical. Examples included BSNP_A09A and BSNP_A09B, BSNP_G01A, BSNP_G01B, BSNP_B02A and BSNP_B02B, and BSNP_C05A and BSNP_C05B (the A and B versions of each haplotype became identical after MHC065 allele removal). Due to this uncertainty and the ambiguous nature of the comparison data, it was not used for further analysis.

The inability to obtain results for MHC065 in Bangladesh chickens could be related to reduced primer compatibility because one or more variable SNPs are present in Bangladesh chickens, compared with other chickens. Because of the high variation in the MHC region, a similar problem might occur when other populations are genotyped in future studies. For example, Nguyen-Phuc et al [26] used 84 SNPs from the aforementioned panel to describe the high MHC haplotype diversity (310 haplotypes) of wild Red Junglefowl populations in Vietnam. Improvements in the comparison of MHC haplotypes would involve either designing new primers for MHC065 amplification or selecting a representative SNP subset from the original MHC panel (90 SNPs) and creating haplotypes that are both consistent and comparable among various native chicken populations.

## CONCLUSION

MHC-B variability in Bangladesh chickens was analyzed using 89 SNPs of the MHC-B SNP panel; the results showed 136 unique haplotypes and only one was shared between populations (HI and NN). However, phylogenetic analysis did not reveal distinct population clusters; it demonstrated three clades containing haplotypes from each population. Therefore, we conclude that MHC-B haplotypes in Bangladesh chickens are unique at the local level and require further investigation to evaluate their relationships with global chicken populations.

## Figures and Tables

**Table 1:** Numbers of individuals used from each population and summary of the obtained haplotypes

Population	Number of individuals	Number of haplotypes		

		Unique	Shared	Total
Aseel	15	20	0	20
Hilly	20	31	1	32
Junglefowl	17	24	0	24
Non-Descript Deshi	20	34	0	34
Naked Neck	19	26	1	27
